# Impact of clinical pharmacist intervention on acute admission unit length of stay

**DOI:** 10.1186/1757-7241-23-S1-A40

**Published:** 2015-07-16

**Authors:** Katrine B Lind, Charlotte A Sørensen, Suheil A Salamon, Marianne Lisby

**Affiliations:** 1 Department of Emergency Medicine, Randers Regional Hospital, Randers, Denmark; 2 Hospital Pharmacy, Central Denmark Region, Denmark; 3 Center of Emergency Medicine Research, Aarhus University Hospital, Aarhus, Denmark

## Background

Quality and efficiency in the Emergency Department (ED) concerns clinicians and administrators worldwide because of an increasing number of patients and a desire for optimizing flow, avoiding crowding, and increasing the quality of the treatment.

For patients referred to an Acute Admission Unit (AAU), which is a sub unit in the ED, the physicians have to obtain a medication history and medication reconciliation that are time consuming. Medication histories obtained by physicians are often incomplete. The objective of the study was to investigate the impact of a clinical pharmacist (CP) intervention on the patients' AAU length of stay (AAU-LOS).

## Methods

The study was a prospective cluster randomised study. Weekdays were randomised for control (standard care) or intervention (standard care plus CP intervention). CP intervention consisted of obtaining a medication history, entering prescriptions into the electronic medication module (EMM), medication reconciliation & review, and a written note in the electronic medical record. The primary outcome measure was AAU-LOS defined as the interval between arrival and discharge or admission to hospital. Secondary outcome measures were physician time spent on medication topics, the number of medications per patient, and for the intervention group number of sources used for obtaining medication history and CP time spent.

## Results

230 and 218 patients were included in the control (n = 62 days) and intervention (n = 64 days) clusters. There were no differences in baseline characteristics between study groups. There was no difference in LOS between the control and intervention group. The un-adjusted LOS was in average 0.9% (95% CI [-7.4; 10%]) longer in the intervention group. The median self-reported physician time spent for medication topics was 7.52 minutes (control group) and 4.29 minutes (intervention group) resulting in an overall reduction of 43.0% (CI: (30.8; 53.0%), p < 0.001). Respectively, 10.1 and 8.8 medications per patient were documented in EMM in the intervention and control group. CPs used on average 12.3 minutes and 3.0 sources to conduct each medication history.

## Conclusion

AAU-LOS was unaffected by CP intervention, although, physicians saved time on medication topics. CPs identified more medications than physicians.

## Trial registration

Clinical Trial Gov: ID-number 1-16-02-379-13.

